# Drug resistant epilepsy driven by *RHEB* gene variants – Current evidence and a novel report of a paedatric case

**DOI:** 10.1016/j.ebr.2026.100856

**Published:** 2026-03-09

**Authors:** Ann-Louise Esserlind, Annie Pedersen, Frida Abel, Tove Hallbook, Colin Reilly, Daniel Nilsson, Tatjana Liakina, Thomas Olsson Bontell

**Affiliations:** a Department of Clinical Sciences, Sahlgrenska Academy, University of Gothenburg, Sweden; b Department of Paediatrics, Queen Silvia Children’s Hospital, Sahlgrenska University Hospital, Member of the ERN EpiCARE, Gothenburg, Sweden; c Department of Clinical Genetics and Genomics, Sahlgrenska University Hospital, Gothenburg, Sweden; d Department of Laboratory Medicine, Institute of Biomedicine, Sahlgrenska Academy, University of Gothenburg, Sweden; e Department of Clinical Neuroscience, Institute of Neuroscience and Physiology, Sahlgrenska Academy, University of Gothenburg, Gothenburg, Sweden; f Department of Neurosurgery, Sahlgrenska University Hospital, Member of the ERN EpiCARE, Gothenburg, Sweden; g Department of Clinical Neurophysiology, Sahlgrenska University Hospital, Member of the ERN EpiCARE, Gothenburg, Sweden; h Department of Clinical Pathology, Sahlgrenska University Hospital, Gothenburg, Sweden; i Department of Physiology, Institute of Neuroscience and Physiology, Sahlgrenska Academy, University of Gothenburg, Gothenburg, Sweden

**Keywords:** Drug resistant focal epilepsy, Pediatric epilepsy, Focal cortical dysplasia, Somatic mosicism, RHEB, mTORopathy, Neurodevelopmental disorders

## Abstract

**Background:**

Malformations of cortical development (MCD), including focal cortical dysplasia type II (FCDII) and hemimegalencephaly (HME), are major causes of drug-resistant focal epilepsy (DRFE). Recent genetic discoveries implicate dysregulation of the mechanistic target of rapamycin (mTOR) signaling pathway as a key driver of abnormal cortical organization and epileptogenesis. Among these, somatic variants in *RHEB* are exceedingly rare but pathogenic, with the recurrent *RHEB* p.Tyr35Leu variant known to hyperactivate mTORC1 signaling.

**Methods:**

We report a paediatric case with an extensive transmantle FCDIIb associated with a somatic *RHEB* p.Tyr35Leu mutation identified through targeted deep sequencing of resected brain tissue. Clinical, radiological and histopathological data were correlated with genetic findings. Additionally, we conducted a comprehensive literature review to contextualize *RHEB*-related neurological disorders within the mTORopathy spectrum and to assess genotype–phenotype correlations.

**Results:**

The identified mosaic *RHEB* variant showed a variant allele frequency (VAF) of 5%, correlating with early-onset, pharmacoresistant epilepsy and extensive cortical dysplasia. Literature synthesis revealed that higher VAF values are associated with greater lesion burden and more severe neurological outcomes. Importantly, somatic *RHEB* variants typically manifest as focal, surgically treatable epilepsies, whereas germline variants produce broader neurodevelopmental and neuropsychiatric phenotypes.

**Conclusions:**

This report expands the clinical and molecular spectrum of *RHEB*-related mTORopathies and emphasizes the diagnostic importance of deep sequencing of brain tissue, as low-level mosaic variants often escape detection in peripheral blood samples. Given that *RHEB* mutations represent potentially druggable mTOR pathway targets, these findings have significant implications for individualized therapeutic strategies and precision epilepsy care.

## Highlights

• We describe a new paediatric case with drug resistant focal epilepsy due to FCDIIb caused by a somatic *RHEB* p.Tyr35Leu variant.
• In a comprehensive integrated case and literature review we delineate clinical patterns in *RHEB*-related mTORopathies.
• Both somatic and germline *RHEB* variants are rare yet represent potentially druggable targets
• This report emphasizes the relevance of comprehensive clinical knowledge of *RHEB*-related mTORopathies for precision epilepsy care.

## 1 Introduction

**Fig. 1:**
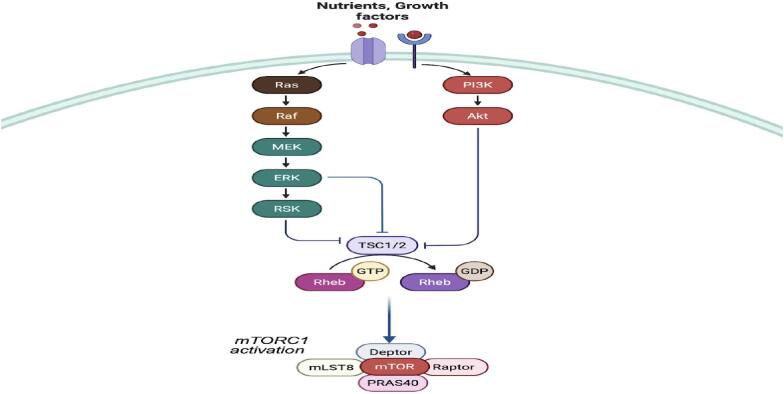
RHEB-dependent activation of the mTORC1 complex. Growth factor driven PI3K–Akt and Ras–ERK signaling inhibits the TSC1/TSC2 complex which keeps RHEB in its active GTP-bound form. Active RHEB directly stimulates mTORC1, which integrates extracellular cues, such as nutrients and growth factors to regulate vital neuronal functions. *Figure created with**Biorender.com**.*

Malformations of cortical development (MCD) represent a diverse group of structural neurodevelopmental anomalies, including focal cortical dysplasia (FCD) and hemimegalencephaly (HME) which arise during neurogenesis in the embryo and are frequently associated with epilepsy and intellectual disability [1], [2], [3], [4]. FCD is a leading cause of drug-resistant focal epilepsy (DRFE) in children and often necessitates surgical intervention [5], [6], [7], [8]. The International League Against Epilepsy (ILAE) classifies FCD based on histopathological features which reflect a spectrum of cortical dysgenesis [9], [10]. Due to the FCDs focal appearance, post-zygotic genetic variants which are strictly found in the brain malformation, so called somatic variants, have warranted further exploration. Indeed, over the last decade, genetic analyses of resected brain tissue from epilepsy surgeries have revealed distinct molecular etiologies, frequently converging on fundamental pathways involved in brain development, cellular proliferation and homeostatic regulation [11], [12], [13], [14], [15]. Among these, the mechanistic target of rapamycin (mTOR) signaling cascade has emerged as a central pathway in the pathogenesis of MCDs, particularly in FCD type II (FCDIIa and b) and HME [16], [17]. The mTOR is a protein serine/threonine kinase at the center of an evolutionarily conserved pathway that drives key aspects of cellular physiology, such as growth, proliferation and survival, through its assembly into two distinct multi-protein complexes: mTORC1 and mTORC2 [18], [19]. Among these, mTORC1 plays a central role in brain development by integrating upstream signaling from nutrients, energy status and growth factors to promote e.g. anabolic processes [20], [21]. Functionally, mTORC1 serves as a critical regulator of neuronal architecture and connectivity, influencing dendritic arborization, axonal elongation, synapse formation and neuronal viability (Fig. 1) [22], [23], [24]. Aberrant activation of the mTOR pathway, either through somatic mutations, germline mutations or both, can lead to abnormal neurodevelopment, focal overgrowth, epilepsy and other neurological disorders collectively termed *mTORopathies* [25], [26], [27], [28]. Hyperactivation of the mTOR pathway, resulting from gain-of-function heterozygous variants in upstream activators encoded by the *PIK3CA, AKT3* and *RHEB* genes collectively account for a substantial proportion of individuals with DRFE associated with FCDII or HME. However, among these genes, somatic variants in *Ras Homolog Enriched in Brain* (*RHEB)* are the rarest occuring [16], [17]. The *RHEB* gene encodes a constitutively active small GTPase that drives mTORC1 signaling and has recently been linked to malformations akin to those observed in other mTORopathies [16], [29], [30], as well as tuberous sclerosis complex(TSC) and neurodevelopmental syndromes [31], [32], [33]. Experimental models in mice suggest that overactive *RHEB* signaling can induce neuronal hypertrophy, dysmorphic cortical architecture, macrocephaly and seizures which is consistent with reported findings in human cases [34], [35]. Seizures associated with somatic *RHEB* variants giving rise to cortical malformations have been described as being refractory to pharmacological treatment and have an early on-set [30]. Surgical resection may offer seizure freedom when the full extent of dysplasia is removed or after hemispherectomy. However, inoperable lesions, incomplete resections or multifocal involvement may limit success in reducing or eliminating seizures after epilepsy surgery. In such scenarios, specific mTORC1 inhibitors known as rapalogs, such as sirolimus and everolimus, could be explored as potential alternatives for targeted treatment [36], [37], [38].

Somatic mutations in *RHEB* are relatively uncommon, representing approximately 3–4.5% of genetic cases of FCDII and HME [16], [17]. Moreover, germline mutations in *RHEB* are exceedingly rarer. To date, only a limited number of studies in humans have described *RHEB*-associated variants resulting in neurological disability consequently leading to a lack of evidence of genotype-phenotype correlations. Here, we report a novel paediatric patient with DRFE who underwent resection of an extensive FCDIIb lesion which showed to harbor a somatic *RHEB* variant. To place our clinical case in a genotype-phenotype context and to summarize the current literature, we performed a comprehensive review of *RHEB* variants linked to neurological and neurodevelopmental disorders.

## 2 Methods and materials

### 2.1 Clinical case report

The paediatric patient was recruited from the Neurology Department at Queen Silvia’s Children’s Hospital, Sahlgrenska University Hospital, Gothenburg, Sweden, as part of a larger research study approved by the Research Ethics Review Board (Research Ethics Committee ID: DN-2024-06222). Written informed consent was obtained from the parents prior to inclusion.

### 2.2 Review of *RHEB* variants associated with neurological and neurodevelopmental disorders in humans

Given the pivotal regulatory function of *RHEB* within the mTOR signaling cascade, which is a pathway essential for cellular proliferation and growth, somatic *RHEB* mutations arising during early embryonic development may result in mosaic overgrowth syndromes, primarily affecting the brain but potentially also involving skin and other organs [39]. The present review compiles existing knowledge on the role of both germline and somatic *RHEB* mutations in neurodevelopmental and neurocutaneous syndromes with a specific focus on epilepsy. Although *RHEB* dysregulation has also been implicated in malignancies and other systemic illnesses [40], [41], [42], such conditions fall outside the scope of this review.

A systematic search was conducted across PubMed, OMIM and Web of Science databases. All studies in English regardless of published date were included based on predefined criteria focused on peer-reviewed case reports, clinical series, and molecular studies documenting neurological phenotypes associated with *RHEB* variants. The following search terms were used: ‘*RHEB* AND neurodevelopmental disorders’; ‘*RHEB* AND epilepsy’; ‘*RHEB* AND neurocutaneous disorders’; ‘*RHEB* AND focal cortical dysplasia OR malformations of cortical development’. The core findings from these publications are summarized in Table 1 and Fig. 6 summarizes the reported variants in a schematic overview of the 184-amino-acid RHEB protein with its functional architecture and the locations of both germline and somatic mutations.

**Table 1:** Review summary of *RHEB* mutations in drug-resistant focal epilepsy (DRFE), neurological and neurodevelopmental disorders (NDDs).

Reference	Gene/protein variant	Variant type	Number of cases/Phenotype
Salinas et al., 2019 [29]	c.104_105delinsTA/p.Tyr35Leu	Somatic (brain tissue) VAF:21%	N = 1; HME, severe neonatal seizures & macrocephaly.
Zhao et al., 2019 [43]	c.104_105delinsTA/p.Tyr35Leu	Somatic (brain tissue) VAF: 5,8–6,4%	N = 1; FCDIIb &focal seizures.
Baldassari et al., 2019 [16]	a. c.104_105delinsTA/p.Tyr35Leub. b.c.119A > T/p.Glu40Val	Somatic (brain tissue) a. VAF: 8.9%; b. VAF: 17.6%	a. N = 1; HME/FCDIIb, neonatal seizures & epidermal naevus syndrome. b. N = 1; Lobar FCDIIb & infantile seizures.
Lee et al., 2021 [30]	c.104_105delinsTA/p.Tyr35Leu	Somatic (brain tissue) VAF: 2.6%	N = 1; Transmantle FCDIIb & sensorimotor seizures.
Lee et al., 2023 [33]	c.104_105delinsTA/p.Tyr35Leu	Somatic (brain tissue) VAF: 13%	N = 1; Drug resistant focal seizures, multiple cortical tubers, SEGA& SEN.
Reijnders et al., 2017 [32]	c.110C > T/p.Pro37Leu c.202 T > C/p.Ser68Pro	Germline (de novo)	a. N = 1; Epilepsy, facial dysmorphisms, severe ID & autism. b. N = 1: Megalencephaly, facial dysmorphisms, severe ID & autism.
Jauss et al., 2022 [44]	c.4C > T/p.Ser16Phe	Germline (de novo)	N = 1; Epilepsy & NDD.
Trujillo-Quintero et al., 2025 [31]	c.71T > C/p.Ile24Thr	Germline (de novo)	N = 1; Epilepsy, facial dysmorphisms & mild ID.

Abbreviations: FCDIIb: Focal Cortical Dysplasia type IIb, HME: Hemimegaloencephaly, ID: intellectual disability, NDD: neurodevelopmental delay, SEGA: subependymal giant cell astrocytoma, SEN: subependymal noduli, TSC: tuberous sclerosis complex, VAF: variant allele frequency.

## 3 Results

### 3.1 Clinical case report

#### 3.1.1 Clinical presentation

The patient is a boy, currently aged 3 years and 4 months, and born to non-consanguineous parents with no family history of epilepsy or other neurological disorders. Pregnancy was uneventful and he was born via cesarean section at gestation week 38 + 4 due to breech presentation. Early psychomotor and neurological development was normal, although mild ptosis of the left eye had been noted since the neonatal period. At 11 months of age, he presented with jerking movements in the left side of the face. Each episode lasted a few seconds and involved twitching around the left eye and corner of the mouth. The child had preserved consciousness during the episodes. Over the course of a week, the frequency of these episodes increased to approximately 5–6 per day. The patient exhibited mildly reduced appetite but was otherwise well-appearing.

**Fig. 2:**
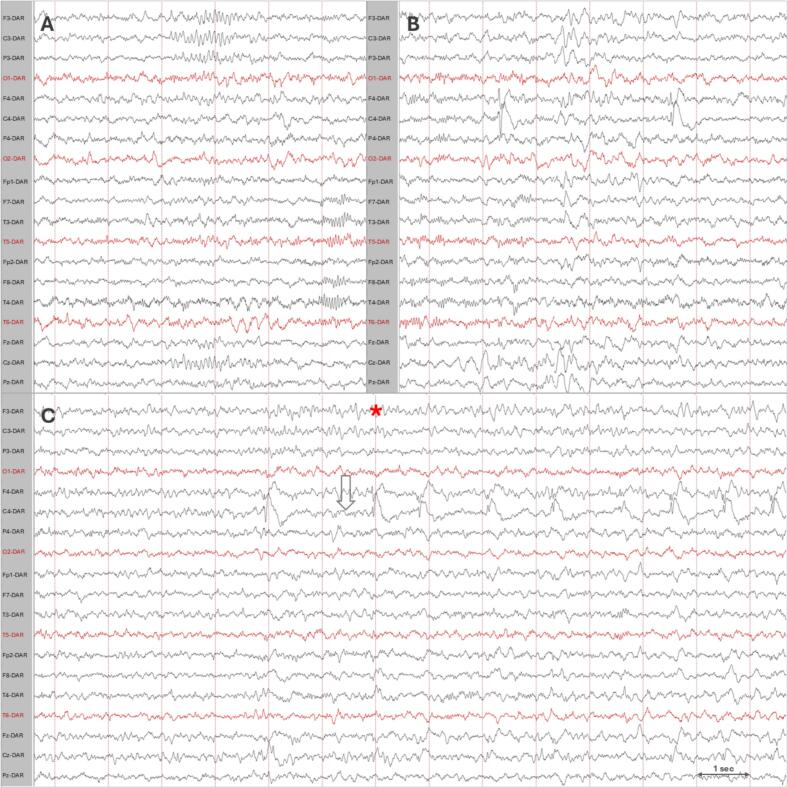
Video electroencephalogram (vEEG): A: Asymmetry in sleep structures showing fewer (poorly formed) sleep spindles in the right hemisphere. B: Focal spike-slow waves discharges in the right paracentral region. C: Typical seizure: low amplitude/high frequency seizure activity evolving to rhythmic spike-slow wave in the right paracentral region (arrow). Onset of clinical symptoms (asterix): squinting of the left eye, left hemifacial contraction. Dynamic average Montage. Filters 5 – 70 Hz.

**Fig. 3:**
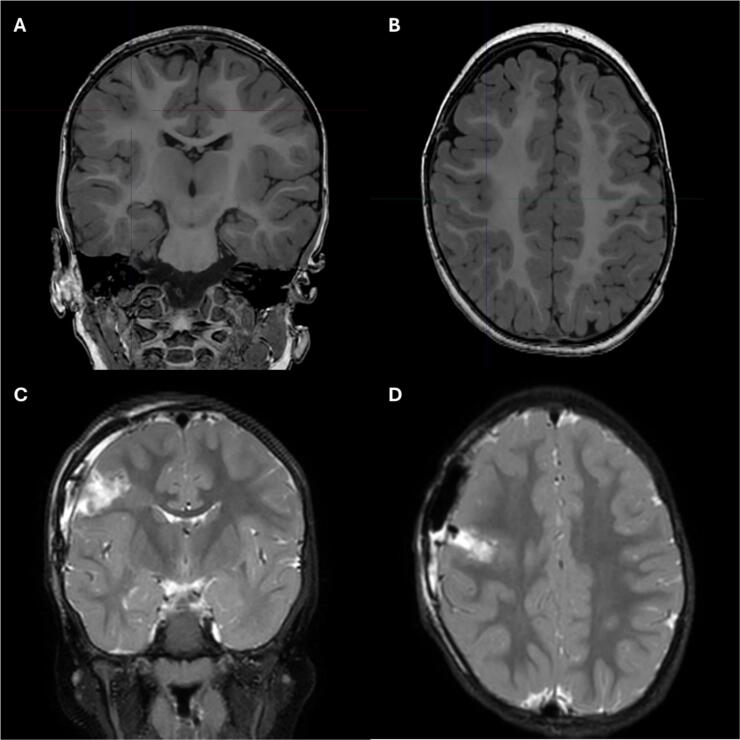
Magnetic resonance imaging (MRi): Coronal (A) and axial (B) T1-weighted 3 T MRI scans showing the dysplasia in the right central region, involving the central sulcus. Postoperative T2-weighted MRI confirmed resection of the dysplasia (C and D).

A paediatric neurologist assessed the patient one week after symptom onset and noted a normal and age-appropriate neurological examination. Standard interictal electroencephalogram (EEG) was done within 2weeks from onset of symptoms and showed a normal background, well-developed sleep structures bilaterally and no epileptiform abnormalities nor focal slowing. Long term video+EEG monitoring two weeks later showed new onset asymmetry in sleep structures, focal epileptiform activity and increased frequency with clusters of up to 100 short seizures withing 30 min occurring up to 40 times per day of highly stereotypical focal seizures in the right central region (Fig. 2). The majority of the short seizures had a duration of 10–25 s and the longest episode lasted 40 s. Imaging with magnetic resonance imaging (MRI) of the brain revealed suspected FCD in the right hemisphere near the central sulcus. The dysplasia exhibited signal changes extending into the underlying white matter, reaching the right frontal horn and showed a transmantle sign. The imaging findings were consistent with FCDII or a tuber (Fig. 3A and 3B). Investigations for tuberous sclerosis complex (TSC) were performed with skin inspection with Woods light, ultrasound of the abdomen, fundoscopy and echocardiography and revealed no abnormalities.

Genetic testing for constitutional (germline) variants was performed by whole genome sequencing on genomic DNA extracted from peripheral blood through library preparation (TrueSeq DNA PCR-free, Illumina) and sequencing (NovaSeq 6000, Illumina) at the Centre for Medical Genomics, at the Sahlgrenska University Hospital, Gothenburg, Sweden. A clinical *in silico* panel including 358 epilepsy-related genes (genes are listed in supplementary material) was analyzed for single nucleotide variants and small insertion/deletions (SNV/indels) and copy number variants (CNV) with negative result.

#### 3.1.2 Treatment

The patient was started on Oxcarbazepine (OXC) 20 mg/kg/day, which led to exacerbated seizure frequency and duration, escalating from 2 to 6 to 20 seizures daily. Due to increased seizure frequency following initiation and dose escalation of OXC, we suspected possible medication associated exacerbation and discontinued this treatment. Although OXC is generally effective for focal onset seizures, paradoxical seizure aggravation has previously been reported in pediatric epilepsy [45], [46], [47]. Subsequent combined therapy with Clobazam 5 mg/day, Lacosamide 16 mg/kg/day and Levetiracetam 60 mg/kg/day stabilized the seizure rates and resulted in temporary improvement but failed to achieve sustained seizure control. Viral illnesses were notable triggers of seizure breakthroughs.

#### 3.1.3 Epilepsy surgery and outcomes

At 22 months of age, the patient underwent epilepsy surgery, including anatomical resection of clearly pathological tissue identified on imaging (Fig. 3C and 3D). Perioperative monitoring of motor function involving the face, upper extremity and lower extremity was performed using short-train cortical and subcortical electrical stimulation. Bipolar stimulation was applied at intensities of 4–10 mA and monopolar stimulation at 10–20 mA. Electrocorticography was employed to monitor for *after discharges* (ADs). No positive motor responses were elicited, and no ADs were recorded. In our experience, as well as that of other centers, motor stimulation in young children may yield false-negative results, likely due to immaturity of the descending corticospinal tract pathways [48].

Postoperatively, the patient achieved sustained seizure freedom. At the 12-month follow-up, he remained seizure-free and demonstrated improved alertness. The surgical intervention resulted in a mild left-sided hemiparesis affecting the arm and leg, which has gradually improved over time.

Given that the cortical dysplasia was in close proximity to, or involved, the primary motor cortex, the occurrence of some degree of postoperative motor deficit was anticipated. The most plausible explanation for the observed post-surgical symptoms is transient disruption of subcortical motor pathways due to surgical manipulation of the corticospinal tract, consistent with the involvement of both upper and lower extremity motor function.

#### 3.1.4 Neurodevelopment

The patient underwent neuropsychological assessment at 18 months of age (four months prior to his surgery). On the Griffiths Developmental Scales – 3rd edition [49] his developmental quotient was 98 (45th percentile), placing him within the average range and indicating no developmental delays. On the Adaptive Behavior Assessment System – Third Edition (ABAS-3) [50] his General Adaptive Composite (GAC) score was 85 (16th percentile) on parent rating, which is at the lower end of the average range. The boy’s adaptive behavior was assessed again when he was 30 months old, thus 8 months after surgery and his GAC on the ABAS-III score was 84 (14th percentile) and thus remaining at the lower end of the average range. The stability of his GAC score at follow-up indicates that, despite surgical intervention, there was no significant decline in adaptive behavior over this period and the boy continued to develop.

#### 3.1.5 Histopathological analysis

**Fig. 4:**
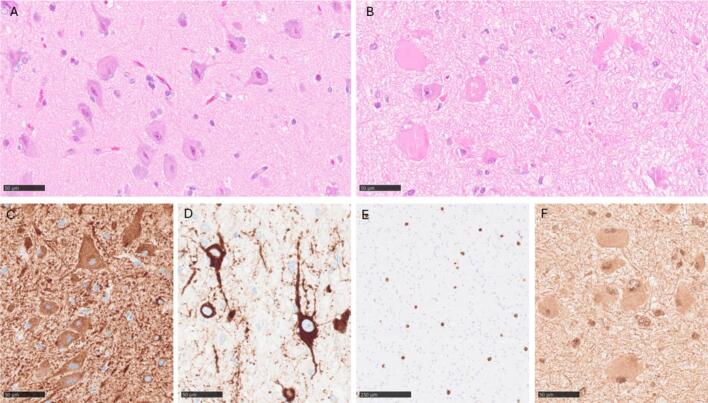
Histology and immunohistochemistry: A. Hematoxylin and eosin staining showing dysmorphic neurons in gray matter. B. Hematoxylin and eosin staining showing balloon cells. C. Immunohistochemical staining showing Map2 positive dysmorphic neurons in gray matter. D. Immunohistochemical staining revealing Map2 positive dysmorphic neurons in white matter. E. Immunohistochemical staining against NeuN highlighting increased number of neurons in white matter. F. Balloon cells in white matter showing positivity for vimentin. Scale bars represent 50 µm with the exception for E where it represents 250 µm.

Formalin-fixed paraffin-embedded (FFPE) resected brain tissue underwent routine histopathological examination, including hematoxylin and eosin (H&E) staining and immunohistochemical (IHC) staining for assessment of tissue architecture and cell morphology (Fig. 4). H&E-stained slides revealed disturbed neuronal layering, dysmorphic neurons in the gray matter and balloon cells especially in the white matter but also in the gray matter (Fig. 4A and 4B). IHC staining with antibodies targeting *GFAP*, *NeuN*, *MAP2*, *NFP* and *Vimentin* was used. *GFAP* staining revealed some gliosis, especially in the white matter. Some dysmorphic neurons were weakly positive for GFAP (staining not shown). Both *NeuN* and *MAP2* staining clarified the disturbed neuronal layering, areas with reduced number of neurons, increased occurrence of neurons in the white matter, a diffuse border between gray- and white matter and highlighted the dysmorphic neurons (Fig. 4C-4E). *NFP* staining showed an increased number of neurons with strong cytoplasmatic positivity (staining not shown). *Vimentin* showed cytoplasmic positivity in balloon cells (Fig. 4F).

#### 3.1.6 Molecular analysis

DNA was extracted from 5 to 10 sections (5 µm) of FFPE-resected brain tissue by QIAamp DNA FFPE Tissue Kit. Library preparation was performed from 100 ng DNA by KAPA Hyper Plus using a custom-made “GMS560” Twist enrichment panel covering single nucleotide variants and small insertion/deletions (SNV/indels) and copy number variants (CNV) in around 560 clinically relevant cancer and overgrowth genes (https://genomicmedicine.se/wp-content/uploads/2023/11/GMS560_synopsis.pdf). The library was sequenced on an Illumina NextSeq 550 sequencer using 2 × 150 (paired-end) at 500-1500x vertical coverage to identify somatic mutations. Targeted sequencing identified a somatic mutation in the *RHEB* gene: NM_005614.4 (*RHEB*): c.104_105delinsTA (p.Tyr35Leu) with a variant allele frequency (VAF) of 5%. This small indel in exon 2 leads to a missense mutation exchanging Tyrosine to Leucine in codon 35 of the RHEB protein, which is a well-conserved region of the GTPase-binding domain. The variant is classified in ClinVar as pathogenic (class 5, ncbi.nlm.nih.gov/clinvar/, ID: 3238630) and has been previously associated with isolated FCDII [30], [43]. In addition, two other variants in the same codon, c.104A > C (p.Tyr35Ser) and c.104A > G (p.Tyr35Cys) are reported in ClinVar and both considered pathogenic (ClinVar Variation IDs 1702651 and 376516). A manual reinspection of the *RHEB* variant (NM_005614.4: c.104_105delinsTA) was performed in the germline WGS data from leucocytes by visualization of the specific genomic position in IGV (g.151188048-151188049, Hg19), but the specific variant could not be detected in any out of 32 sequencing reads. Hence, the *RHEB* variant was considered as somatic.

#### 3.1.7 Functional analysis by IHC

**Fig. 5:**
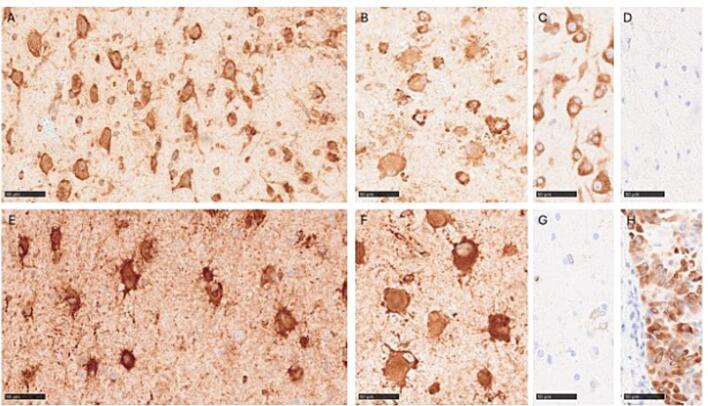
S6 and pS6 (Ser235/236) immunohistochemistry: A. Strong S6 expression in dysmorphic neurons. B. Strong S6 expression in balloon cells. C. Strong S6 expression in non-dysmorphic neocortical neurons in control tissue. D. No positivity in control stain (omitted antibody). E. Strong pS6 expression in dysmorphic neurons. F. Strong pS6 expression in balloon cells. G. No pS6 expression in non-dysmorphic neocortical neurons in control tissue. H. Strong pS6 expression in positive control (lung adenocarcinoma). Scale bars represent 50 µm.

To verify the activation of the mTOR signaling pathway in affected brain tissue, IHC staining for ribosomal protein S6 and phosphorylation of Ser235/236 in S6 (pS6), a downstream biomarker of mTOR pathway activation was performed according to previously described methods [51]. Dysmorphic neurons and balloon cells showed strong expression of S6 (Fig. 5A and 5B). They alsoshowed strong positivity for pS6, consistent with mTOR activation (Fig. 5E and 5F). Non-dysmorphic neocortical neurons in control tissue also showed strong expression for S6 but were negative for pS6 (Fig. 5C and 5G).

**Fig. 6:**
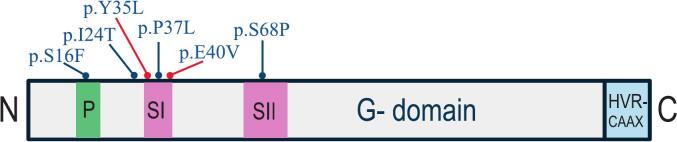
Schematic presentation and localization of pathogenic DNA-variants in *RHEB*: The *RHEB* gene located on chromosome 7 encodes a 184-amino-acid small GTPase protein that localizes to lysosomal membranes via its C-terminal CAAX motif. Its G-domain (residues 1–169) binds GTP and allosterically activates mTORC1 in response to nutrients and growth factors, promoting phosphorylation of downstream effectors S6 kinase and 4E-BP1. Domains of the GTP-binding region are as follows; P-loop (residues 10–20), Switch I region (SI, residues 33–41), switch II region (SII, residues 63–79). The 15 remaining C-terminal residues make up a highly variable region (HVR) ending in a CAAX motif [52], [53]. Blue dots indicate germline mutations and red dots mark somatic mutations reviewed in this report. Key functions and regulatory interactions summarized from UniProt and referenced studies. [16], [29], [30], [31], [32], [33], [43], [44], [54], [55], [56], [57]^.^ (For interpretation of the references to colour in this figure legend, the reader is referred to the web version of this article.)

### 3.2 Review of *RHEB* variants associated with neurological and neurodevelopmental disorders

#### 3.2.1 RHEB variants in epilepsy and focal cortical dysplasia

The implication of *RHEB* variants in cortical malformations was first recognized through high-depth sequencing studies of drug-resistant epilepsy patients with FCDII or HME. In 2019, Salinas *et al.* identified a low-level somatic mutation in *RHEB* (c.104_105delinsTA, encoding p.Tyr35Leu) in a child with HME and intractable epilepsy [29]. This provided the first evidence linking *RHEB* to human cortical dysgenesis. Shortly thereafter, Zhao *et al.* reported the same brain-specific *RHEB* variant, in a patient with refractory epilepsy and FCDII [43]. In this patient, the *RHEB*-mutant neurons showed markedly increased phosphorylated S6 (pS6) staining, indicating hyperactive mTOR signaling in the dysplastic tissue. Zhao *et al.* also conducted in-vivo experiments confirming the mutation’s pathogenicity by performing in-utero electroporation of *RHEB* p.Tyr35Leu into developing mouse brain which then induced enlarged, dysmorphic neurons, cortical lamination defects, EEG abnormalities and spontaneous seizures [43], These findings essentially mimicked symptoms observed in humans with FCDII. [10], [43], [58]. Treatment with the mTORC1 inhibitor, Rapamycin, in this mouse model suppressed the epileptic activity thus supporting that the *RHEB* p.Tyr35Leu variant causes epilepsy via mTOR hyperactivation.

Subsequently, additional patients with somatic *RHEB* variants have been described. A 2019 multi-center cohort study of 80 FCDII and HME cases found *RHEB* variants in a small fraction of patients (2 cases), consistent with *RHEB* being less frequently implicated in mTORopathies compared to *MTOR* gene itself [16].

These findings support a pathogenic role of the *RHEB* variants, as all FCDII samples with somatic *RHEB* mutations have shown mTORC1 activation on histopathology (dense pS6-positive dysmorphic neurons and balloon cells), although mTOR activation alone cannot definitively establish causality. In fact, a recent study further links mTOR hyperactivation to both structural and intrinsic membrane abnormalities in *RHEB*-associated cortical malformations by modelling a hyperactive Rheb–mTOR signaling in mice by in-utero electroporation of constitutively active Rheb (Rheb^S16H^) into layer 2/3 pyramidal neurons. In this setting, Rheb-expressing neurons showed not only cytomegaly but also increased dendritic complexity and altered hyperpolarization activated cyclic nucleotide gated (HCN) currents, with a prominent contribution of HCN4. HCN channels carry an inward cation current activated at hyperpolarized membrane potentials (termed I_h) that depolarizes neurons and helps set their baseline firing rate. Enhanced HCN4-mediated I_h, rather than increased synaptic excitation, appeared to be a major driver of the elevated firing of these dysmorphic pyramidal neurons [59].

Interestingly, all reported *RHEB* mutations in FCDII and HME to date have been missense changes predicted to be gain-of-function variants [16], [60], [61]. The most recurrent *RHEB* variant associated with FCDII is the p.Tyr35Leu [16], [29], [30], was also confirmed in our patient case. Another reported FCDII-linked variant is the *RHEB* p.Glu40Val, identified in a child with a FCDIIb lesion [30]. Both the Tyr35 and Glu40 substitutions are localized in the switch I region of the G-domain which essentially lock RHEB in a GTP-bound constitutively active state, thereby increasing the amount of active RHEB [42], [62] leading to hyperactivation of mTORC1 (Fig. 6). Clinically, patients with somatic *RHEB* mutations appear to have very early seizure onset and severe epilepsy phenotypes. This aligns with the hypothesis that *RHEB* mutations likely occur early in embryonic brain development, leading to a large burden of dysplastic and hyperexcitable neurons [30], [43], [63]. Furthermore, there is a positive correlation between the somatic mutation load and lesion size. Low-level *RHEB* mosaicism (<3% VAF) may produce a confined transmantle FCDII, while higher mutation loads (>15% VAF) seem to drive diffuse hemispheric enlargement [30]. In terms of treatment, focal lesions with somatic *RHEB*-variants have proven to be amenable to epilepsy surgery, which has shown to be curative if the entire malformation is removed.

In addition to focal cortical malformations, *RHEB* mutations have shown to also underlie TSC which is a neurocutaneous syndrome caused by mutations in the mTOR pathway [64], [65]. Typically, TSC is caused by classical germline (heterozygous) variants or post-zygotic mosaic mutations in *TSC1* or *TSC2* genes, leading to multi-organ hamartomas including cortical tubers, subependymal nodules, renal angiomyolipomas, cutaneous lesions and the majority of individuals develop epilepsy at an early age. In 2023, Lee et al. reported a child with drug-resistant infantile spasms, multiple cortical tubers on MRI, subependymal giant cell astrocytoma (SEGA) and skin lesions consistent with TSC but where genetic testing of blood was negative for the typical *TSC1* or *TSC2* variants [33]. Deep sequencing of the affected brain tissue revealed a mosaic *RHEB* c.104_105delinsTA (p.Tyr35Leu) mutation at approximately 14% VAF in a resected tuber. The *RHEB*-mutant cells showed strong pS6 immunoreactivity suggestive of mTORC1 activation like in typical TSC tubers. The recurrent *RHEB* variant described by Lee et al. was identical to that previously observed in paediatric cases of FCDII and HME [16], [29], [30], [43], as well as in our present case. Lee et al. further proposed that somatic *RHEB* mutations may phenocopy TSC and that *RHEB* should be considered a rare third TSC gene (TSC3) in patients with a TSC phenotype who lack pathogenic variants in TSC1 or TSC2 [33].

#### 3.2.2 Germline RHEB variants in neurological and neurodevelopmental disorders

Germline *RHEB* mutations appear to be rare and manifest as a form of congenital mTORopathy characterized by syndromic developmental disorders including brain overgrowth causing megalocephaly, neurodevelopmental delay and epilepsy. To date, only a few germline *RHEB* variants have been reported in neurodevelopmental disorders. A study from 2017 by Reijnders and colleagues further lends support to the fact that germline *RHEB* variants are associated with megalocephaly and intellectual disability [32]. In line with these findings, Jauss et al., described an additional individual harboring a *de novo* heterozygous *RHEB* variant who presented with global developmental delay, epilepsy, macrocephaly and structural brain abnormalities. Although the reported variant by Jauss et al. was initially classified as a variant of uncertain significance (VUS), it is still listed as pathogenic in the Human Gene Mutation Database (HGMD), further supporting the implication of *RHEB* in syndromic mTORopathies [44]. A more recent publication by Trujillo-Quintero *et al.* described a patient with global developmental delay, intellectual disability, epilepsy and subtle dysmorphic features who carried a *de novo* heterozygous missense variant in *RHEB* (c.71T > C, p.Ile24Thr) [31]. This variant affects the N-terminal region of *RHEB* and was deemed likely pathogenic as an mTORopathy-variant. The child’s clinical presentation overlapped with the TSC phenotype but did not have overt cortical tubers or FCDs but the symptomatology rather represented a more diffuse neurological dysfunction.

## 4 Discussion

In this study, we describe an infant presenting with DRFE stemming from FCDIIb, attributed to a *RHEB* p.Tyr35Leu mutation identified in surgically resected brain tissue. Our findings align with previously documented paediatric cases with FCDIIb and HME exhibiting early-onset, pharmacoresistant epilepsy and pronounced cortical malformations linked to the same *RHEB* variant [16], [29], [30], [33], [43].

Consistent with prior results, these somatic *RHEB* mutations are confined to lesional brain tissue and often elude detection through standard peripheral blood sequencing and underlines the importance of deep, targeted genetic profiling of resected tissue to secure an accurate molecular diagnosis [15], [29], [33], [66]. From a clinical counseling perspective, confirming a somatic mosaicism provides reassurance regarding the low recurrence risk for siblings and likely minimal systemic complications.

While the published literature on neurological disorders linked to *RHEB* mutations is limited and thereby hinders broader generalizations, our small yet comprehensive review of genotype-phenotype comparisons suggests that somatic *RHEB* variants, particularly the recurrent p.Tyr35Leu variant, apparently drive focal cortical malformations. These mosaic variants were characterized by pronounced mTOR pathway (mTORC1) hyperactivation as demonstrated by elevated phospho-S6 immunoreactivity in our specimen as well as in previous reports [16], [33], [43]. Clinically, the somatic variants in *RHEB* lead to early-onset, frequent and seizure medication–resistant seizures. Moreover, previously published assessments indicate that higher variant allele frequency (VAF) correlates with more extensive dysplasia and greater neurological impairment [16], [30]. In contrast, rare germline *RHEB* variants tend to manifest broader neurodevelopmental phenotypes, including intellectual disability, autism spectrum disorder, behavioural disturbances, macrocephaly and more diffuse rather than focal neurological involvement [31], [32], [44]. Neurodevelopmental comorbidities also seem to be more prevalent among individuals with germline *RHEB* mutations, aligning with broader observations within the mTORopathy spectrum, where systemic mTOR pathway dysregulation may contribute significantly to psychiatric and cognitive dysfunction [64], [67], [68], [69], [70]. Nevertheless, given the scarcity of published reports, these genotype-phenotype correlations must be interpreted with caution and further reports are necessary to substantiate these preliminary findings.

Distinguishing somatic from germline *RHEB* mutations is essential for guiding treatment and prognostication. Surgical resection remains the treatment of choice for FCDIIb, as demonstrated in our patient (who is seizure-free over a year after epilepsy surgery) as well as in previous studies. [16], [29], [30] Given the great postoperative seizure control in our case and the lack of major developmental comorbidity, we did not pursue adjuvant mTORC1-targeting therapy but rather focused on tapering the child’s anti-seizure medications. Nevertheless, in selected scenarios with incomplete resection, multifocal lesions, postsurgical seizure recurrence and perhaps in children with marked developmental impairment, mTOR-pathway inhibitors (e.g., rapalogs such as everolimus and sirolimus) may be considered on a case-by-case basis. Any such prescription should be done cautiously, as clinical data remain inconsistent and much of the evidence for *RHEB*-driven epileptogenesis and rapalog sensitivity comes from experimental models rather than patient cohorts. Therefore, thorough risk–benefit evaluation and harmonized outcome reporting are warranted.

A recent phase II, prospective, crossover, placebo-controlled trial by Kim et al., evaluated everolimus in patients with pathologically confirmed FCDII. Although the overall cohort did not show significant seizure reduction compared to placebo, a subset of patients harbouring somatic *MTOR* pathogenic variants or with no identified genetic abnormalities achieved seizure freedom during everolimus treatment, while patients with other genetic backgrounds did not [71]. This finding is especially relevant to *RHEB*-associated DRFE, as RHEB functions upstream of mTOR within the same signaling pathway, suggesting that patients with somatic *RHEB* mutations may similarly benefit from mTORC1-targeted therapy [43], [72], [73]. To further support this notion, Zhao et al. used rapamycin (sirolimus) to rescue dysmorphic neurons and demonstrated seizure suppression in mouse models harboring the *RHEB* p.Tyr35Leu mutation [43]. Moreover, although mTOR inhibitors have demonstrated effectiveness in managing TSC-related manifestations such as DRFE, their influence on neuropsychological outcomes remains unclear, as current evidence suggests limited or no beneficial impact on cognitive and behavioural deficits in children older than two years [72], [73], [74], [75]. However, improvements in cognitive and behavioural outcomes remain modest when treatment initiation is started after infancy. These observations raise the possibility of a critical therapeutic window during early neurodevelopment, before the onset of seizures or infantile spasms, when mTOR modulation may be most impactful. An ongoing randomized clinical trial by Driedger et al. aims to explore this premise [76], and if the outcome proves to be favorable, the findings may hold clinical relevance beyond TSC, potentially benefiting also young children with germline *RHEB* mutations characterized by significant neurodevelopmental impairments.

Emerging experimental strategies also offer potential future therapeutical avenues including localized drug delivery techniques designed to achieve targeted intralesional inhibition of RHEB-mediated mTORC1 hyperactivation while minimizing systemic adverse effects may hold significant promise. Additionally, innovative gene therapy strategies using viral vectors to deliver modified potassium channels aimed at reducing seizure activity, as proposed by Barbanoj et al., may also offer potential breakthroughs in treating conditions associated with somatic *RHEB* mutations [37], [38], [77].

In conclusion, as advances in genetic research continue to elucidate the molecular underpinnings of DRFE and related neurological disorders, comprehensive clinical characterisation of affected individuals remains critical for guiding therapeutic decisions and refining prognostic assessments. By following up on previously reported variants and integrating data from a novel case with existing literature, our study contributes to a more nuanced understanding of the genotype-phenotype correlations in *RHEB*-associated mTORopathies. These findings highlight the importance of combining molecular diagnostics with detailed clinical evaluation to enhance diagnostic precision, inform personalized management strategies and hopefully improve patient outcomes. Continued research into the expanding spectrum of genotype-phenotype relationships and emerging therapeutic interventions will be important for optimizing care in this group of rare neurological disorder.

## Ethical statement

The publication of this case report is part of a larger study and has been approved by the Research Ethics Committee in Sweden, approval number: 2024-06222-01. Written informed consent for publication of clinical details and accompanying images was obtained from the child’s parents. Identifying information has been removed to protect the patient’s privacy and all data were handled in compliance with applicable data-protection regulations, including GDPR and stored in secure, access-restricted systems. No interventions outside standard clinical care were undertaken for the purpose of this report.

## CRediT authorship contribution statement

**Ann-Louise Esserlind:** Writing – review & editing, Writing – original draft, Visualization, Project administration, Methodology, Data curation, Conceptualization. **Annie Pedersen:** Writing – review & editing, Writing – original draft, Methodology, Data curation. **Frida Abel:** Writing – review & editing, Writing – original draft, Visualization, Data curation, Conceptualization. **Tove Hallbook:** Writing – review & editing, Writing – original draft, Visualization. **Colin Reilly:** Writing – review & editing, Writing – original draft, Visualization, Data curation. **Daniel Nilsson:** Writing – review & editing, Writing – original draft, Project administration, Data curation. **Tatjana Liakina:** Writing – review & editing, Writing – original draft, Data curation. **Thomas Olsson Bontell:** Writing – review & editing, Writing – original draft, Visualization, Project administration, Data curation, Conceptualization.

## Funding

This report was supported by a research grant from the Research Fund for Epilepsy, Föreningen Margarethahemmet in Sweden.

## Declaration of competing interest

The authors declare that they have no known competing financial interests or personal relationships that could have appeared to influence the work reported in this paper.
