# Supplementary material for: Drug resistant epilepsy driven by RHEB gene variants – Current evidence and a novel report of a paedatric case
Source: Epilepsy Behav Rep. 2026 Mar 9;34:100856. doi: 10.1016/j.ebr.2026.100856 (PMC13049671; doi:10.1016/j.ebr.2026.100856)
Supplement: Supplementary Data 1 [file mmc1.docx]

358 genes included in the WGS in silico panel for epilepsy

| AARS |
| --- |
| ABAT |
| ABCC8 |
| ACTB |
| ACY1 |
| ADAR |
| ADCY5 |
| ADRA2B |
| ADSL |
| AFG3L2 |
| AGTR2 |
| ALDH5A1 |
| ALDH7A1 |
| ALG1 |
| ALG11 |
| ALG13 |
| ALG3 |
| ALG6 |
| AMACR |
| AMT |
| AP3B2 |
| AP4S1 |
| APOPT1 |
| ARHGEF9 |
| ARID1B |
| ARV1 |
| ARX |
| ASAH1 |
| ASL |
| ASNS |
| ATP13A2 |
| ATP1A2 |
| ATP1A3 |
| ATP6AP2 |
| ATP7A |
| ATRX |
| AUTS2 |
| BOLA3 |
| BRAT1 |
| BRWD3 |
| BSCL2 |
| BTD |
| CACNA1A |
| CACNA1E |
| CACNA2D1 |
| CACNA2D2 |
| CAD |
| CASK |
| CDKL5 |
| CERS1 |
| CHD2 |
| CHRNA2 |
| CHRNA4 |
| CHRNA7 |
| CHRNB2 |
| CLDN16 |
| CLDN19 |
| CLN3 |
| CLN5 |
| CLN6 |
| CLN8 |
| CNKSR2 |
| CNNM2 |
| CNTN2 |
| CNTNAP2 |
| COL4A1 |
| COL4A3BP |
| COQ2 |
| COQ4 |
| COQ8A |
| CPA6 |
| CPS1 |
| CPT2 |
| CRADD |
| CSTB |
| CTSD |
| CTSF |
| CUL4B |
| D2HGDH |
| DCX |
| DDC |
| DEAF1 |
| DENND5A |
| DEPDC5 |
| DLAT |
| DNAJC5 |
| DNM1 |
| DNM1L |
| DOCK7 |
| DOLK |
| DPAGT1 |
| DPM1 |
| DPM2 |
| DPYD |
| DYNC1H1 |
| DYRK1A |
| EEF1A2 |
| EGF |
| EHMT1 |
| EIF2S3 |
| EPM2A |
| FA2H |
| FARS2 |
| FGD1 |
| FGF12 |
| FLNA |
| FOLR1 |
| FOXG1 |
| FOXRED1 |
| FRRS1L |
| FXYD2 |
| GABRA1 |
| GABRB1 |
| GABRB2 |
| GABRB3 |
| GABRD |
| GABRG2 |
| GAL |
| GAMT |
| GATM |
| GCK |
| GCSH |
| GLDC |
| GLRA1 |
| GLRB |
| GLUD1 |
| GNAO1 |
| GNB1 |
| GNB5 |
| GOSR2 |
| GPC3 |
| GPHN |
| GRIA3 |
| GRIN1 |
| GRIN2A |
| GRIN2B |
| GRIN2D |
| GRN |
| GUF1 |
| HADH |
| HCN1 |
| HDAC4 |
| HECW2 |
| HLCS |
| HNRNPH2 |
| HNRNPU |
| HSD17B10 |
| HSD17B4 |
| IDH2 |
| IER3IP1 |
| IFIH1 |
| IQSEC2 |
| ITPA |
| JAM3 |
| KANSL1 |
| KCNA1 |
| KCNA2 |
| KCNB1 |
| KCNC1 |
| KCNH1 |
| KCNJ10 |
| KCNJ11 |
| KCNMA1 |
| KCNQ2 |
| KCNQ3 |
| KCNT1 |
| KCTD7 |
| KDM5C |
| KIF1A |
| KIF5A |
| KMT2A |
| KPTN |
| LGI1 |
| LIAS |
| LMNB2 |
| MAGEL2 |
| MBD5 |
| MBOAT7 |
| MDH2 |
| MECP2 |
| MED12 |
| MED17 |
| MEF2C |
| MFF |
| MFSD8 |
| MLC1 |
| MOCS1 |
| MOCS2 |
| MPDU1 |
| MTHFR |
| MTOR |
| NACC1 |
| NAPB |
| NDUFA1 |
| NDUFA11 |
| NDUFAF1 |
| NDUFAF2 |
| NDUFAF3 |
| NDUFAF4 |
| NDUFAF5 |
| NDUFB3 |
| NDUFB9 |
| NDUFS1 |
| NDUFS2 |
| NDUFS3 |
| NDUFS4 |
| NDUFS6 |
| NDUFV1 |
| NDUFV2 |
| NECAP1 |
| NEDD4L |
| NEXMIF |
| NGLY1 |
| NHLRC1 |
| NPRL2 |
| NPRL3 |
| NR2F1 |
| NRXN1 |
| NUBPL |
| OFD1 |
| OPHN1 |
| OTUD6B |
| PAFAH1B1 |
| PAK3 |
| PC |
| PCDH12 |
| PCDH19 |
| PDHA1 |
| PDHB |
| PDP1 |
| PDX1 |
| PET100 |
| PEX1 |
| PEX10 |
| PEX12 |
| PEX13 |
| PEX14 |
| PEX16 |
| PEX19 |
| PEX26 |
| PEX3 |
| PEX5 |
| PEX6 |
| PGAP3 |
| PHF6 |
| PHGDH |
| PIGA |
| PIGG |
| PIGN |
| PIGO |
| PIGQ |
| PIGT |
| PIGV |
| PIGW |
| PLA2G6 |
| PLCB1 |
| PLP1 |
| PLPBP |
| PMM2 |
| PNKP |
| PNPO |
| POLG |
| PPP2R1A |
| PPT1 |
| PQBP1 |
| PRDM8 |
| PRICKLE1 |
| PRICKLE2 |
| PRRT2 |
| PRUNE1 |
| PURA |
| PYCR2 |
| QARS |
| RAB39B |
| RARS2 |
| RBSN |
| RELN |
| RFT1 |
| RNASEH2A |
| RNASEH2B |
| RNASEH2C |
| ROGDI |
| RORB |
| RPS6KA3 |
| RRM2B |
| RUSC2 |
| SAMHD1 |
| SCARB2 |
| SCN1A |
| SCN1B |
| SCN2A |
| SCN3A |
| SCN8A |
| SCN9A |
| SEPSECS |
| SERPINI1 |
| SHANK3 |
| SIK1 |
| SLC12A5 |
| SLC13A5 |
| SLC16A1 |
| SLC19A3 |
| SLC1A2 |
| SLC1A4 |
| SLC25A1 |
| SLC25A12 |
| SLC25A15 |
| SLC25A22 |
| SLC2A1 |
| SLC35A2 |
| SLC39A8 |
| SLC6A1 |
| SLC6A8 |
| SLC9A6 |
| SMARCA2 |
| SMC1A |
| SMS |
| SPATA5 |
| SPTAN1 |
| SRPX2 |
| ST3GAL3 |
| ST3GAL5 |
| STAMBP |
| STRADA |
| STX1B |
| STXBP1 |
| STYXL1 |
| SUMF1 |
| SUOX |
| SYN1 |
| SYNGAP1 |
| SYNJ1 |
| SYP |
| SZT2 |
| TANGO2 |
| TBC1D24 |
| TBCD |
| TBCE |
| TCF4 |
| TDP2 |
| TPP1 |
| TREX1 |
| TRIM8 |
| TRPM6 |
| TSC1 |
| TSC2 |
| TSEN54 |
| TUBA1A |
| TUBB2A |
| TUBG1 |
| UBA5 |
| UBE3A |
| UNC80 |
| WDR45 |
| WWOX |
| ZBTB18 |
| ZEB2 |
| ZNHIT3 |
